# TBX3 is dynamically expressed in pancreatic organogenesis and fine-tunes regeneration

**DOI:** 10.1186/s12915-023-01553-x

**Published:** 2023-03-20

**Authors:** Michael Karl Melzer, Silvia Schirge, Johann Gout, Frank Arnold, Dharini Srinivasan, Ingo Burtscher, Chantal Allgöwer, Medhanie Mulaw, Friedemann Zengerling, Cagatay Günes, Heiko Lickert, Vincent M. Christoffels, Stefan Liebau, Martin Wagner, Thomas Seufferlein, Christian Bolenz, Anne M. Moon, Lukas Perkhofer, Alexander Kleger

**Affiliations:** 1 Clinic of Internal Medicine I, Ulm University Hospital, Ulm, 89081 Germany; 2 Clinic of Urology, Ulm University Hospital, Ulm, 89081 Germany; 3 Institute of Molecular Oncology and Stem Cell Biology, Ulm University Hospital, Ulm, 89081 Germany; 4 Institute of Diabetes and Regeneration Research, Helmholtz Diabetes Center, Helmholtz Zentrum München, 85764 Neuherberg, Germany; 5 Institute of Stem Cell Research, Helmholtz Zentrum München, 85764 Neuherberg, Germany; 6 German Center for Diabetes Research (DZD), 85764 Neuherberg, Germany; 7 Unit for Single-cell Genomics, Ulm University, 89081 Ulm, Germany; 8 Chair of b-Cell Biology, Technische Universität München, School of Medicine, Klinikum Rechts der Isar, 81675 München, Germany; 9 Department of Medical Biology, Amsterdam Cardiovascular Sciences, Amsterdam UMC, University of Amsterdam, Meibergdreef 15, 1105AZ Amsterdam, The Netherlands; 10 Institute of Neuroanatomy & Developmental Biology (INDB), Eberhard Karls University Tübingen, Österbergstrasse 3, 72074 Tübingen, Germany; 11 Department of Molecular and Functional Genomics, Weis Center for Research, Geisinger Clinic, Danville, PA USA; 12 Department of Human Genetics (adjunct), University of Utah, Salt Lake City, UT USA; 13 The Mindich Child Health and Development Institute, Hess Center for Science and Medicine at Mount Sinai, New York, NY USA; 14 Core Facility Organoids, Ulm University, 89081 Ulm, Germany

**Keywords:** Pancreatic development, TBX3, Acute pancreatitis, Organ regeneration, Embryonic development

## Abstract

**Background:**

The reactivation of genetic programs from early development is a common mechanism for injury-induced organ regeneration. T-box 3 (TBX3) is a member of the T-box family of transcription factors previously shown to regulate pluripotency and subsequent lineage commitment in a number of tissues, including limb and lung. TBX3 is also involved in lung and heart organogenesis. Here, we provide a comprehensive and thorough characterization of TBX3 and its role during pancreatic organogenesis and regeneration.

**Results:**

We interrogated the level and cell specificity of TBX3 in the developing and adult pancreas at mRNA and protein levels at multiple developmental stages in mouse and human pancreas. We employed conditional mutagenesis to determine its role in murine pancreatic development and in regeneration after the induction of acute pancreatitis. We found that *Tbx3* is dynamically expressed in the pancreatic mesenchyme and epithelium. While *Tbx3* is expressed in the developing pancreas, its absence is likely compensated by other factors after ablation from either the mesenchymal or epithelial compartments. In an adult model of acute pancreatitis, we found that a lack of *Tbx3* resulted in increased proliferation and fibrosis as well as an enhanced inflammatory gene programs, indicating that *Tbx3* has a role in tissue homeostasis and regeneration.

**Conclusions:**

TBX3 demonstrates dynamic expression patterns in the pancreas. Although TBX3 is dispensable for proper pancreatic development, its absence leads to altered organ regeneration after induction of acute pancreatitis.

**Supplementary Information:**

The online version contains supplementary material available at 10.1186/s12915-023-01553-x.

## Summary statement

TBX3 shows switching expression patterns during embryonic development in the pancreas and leads to fine-tuning of regeneration from acute pancreatitis via limiting proliferation and fibrosis during regeneration.

## Background

The family of T-box transcription factors includes five subfamilies and comprises 17 genes in both humans and mice. They all share highly conserved “T” DNA-binding domains and are essential during embryonic development and tissue homeostasis [1–4].

*TBX3*, along with *TBX2*, *TBX4*, and *TBX5*, belongs to the *TBX2*-family [1, 2]. Heterozygous mutations in *TBX3* lead to the malformation of multiple structures resulting in ulnar-mammary syndrome in humans [5]. Accordingly, homozygotes for hypomorphic mutations in *Tbx3* in murine embryos and adults resulted in lethal maldevelopment of the heart and cardiac arrhythmias [4–6].

TBX3 has multiple molecular functions [7] and participates in transcription factor networks that guide organ patterning in multiple tissues. TBX3 and TBX2 are closely entangled in other regulatory programs, such as Wnt and Hedgehog signaling, and both are essential for proper limb [8], lung [9], and ureter development [10]. Interestingly, redundancy between *Tbx3* and *Tbx2* to compensate for each other has been reported in several organs [9, 10].

Finally, TBX3 is not only critically involved in pluripotency maintenance but also in lineage-specific exit from the pluripotency circuitry in mouse embryonic stem cells [11–17]. *TBX3* is also a putative inhibitor of pancreatic lineage entry in differentiating human pluripotent stem cells [18]. Interestingly, *TBX3* was associated with higher aggressiveness in pancreatic cancer via its ability to (i) promote angiogenesis and (ii) induce cancer stem cell properties [19]. Such stem cell-related features may also become relevant during tissue regeneration after organ injury.

Common mechanisms and similar genetic programs between stem cell induction, organ homeostasis, and regeneration from injury have been identified for the pancreas [20, 21]. Among others, augmented Hedgehog, Notch, and Wnt/b-catenin signaling were delineated as mandatory for regeneration in mice after experimental induction of pancreatitis [21–24]. Interestingly, *Tbx3* interacts with certain parts of the Wnt and Hedgehog signaling pathways [8–10].

To investigate the functional roles of *Tbx3* during pancreatic development, adult pancreatic homeostasis, and pancreatic regeneration after injury, we here re-analyzed publicly available single-cell transcriptomic data sets and employed a *Tbx3*^*Venus*^ reporter system as well as a compartment-specific *Tbx3* knockout mouse model and a human knockdown induced pluripotent stem cell (iPSC) differentiation model. While TBX3 is expressed in both mesenchyme and epithelium during embryonic development, its deletion does not significantly alter organogenesis. However, loss of *Tbx3* leads to overshoot proliferation of acinar cells, accumulation of fibrosis, and enhanced inflammatory stimuli, Il6-Jak-Stat3, and acinar cell-specific NF-κB signaling during pancreatitis.

## Results

### TBX3 is dynamically expressed during pancreatic organogenesis and in adult pancreatic stellate cells

**Fig. 1:**
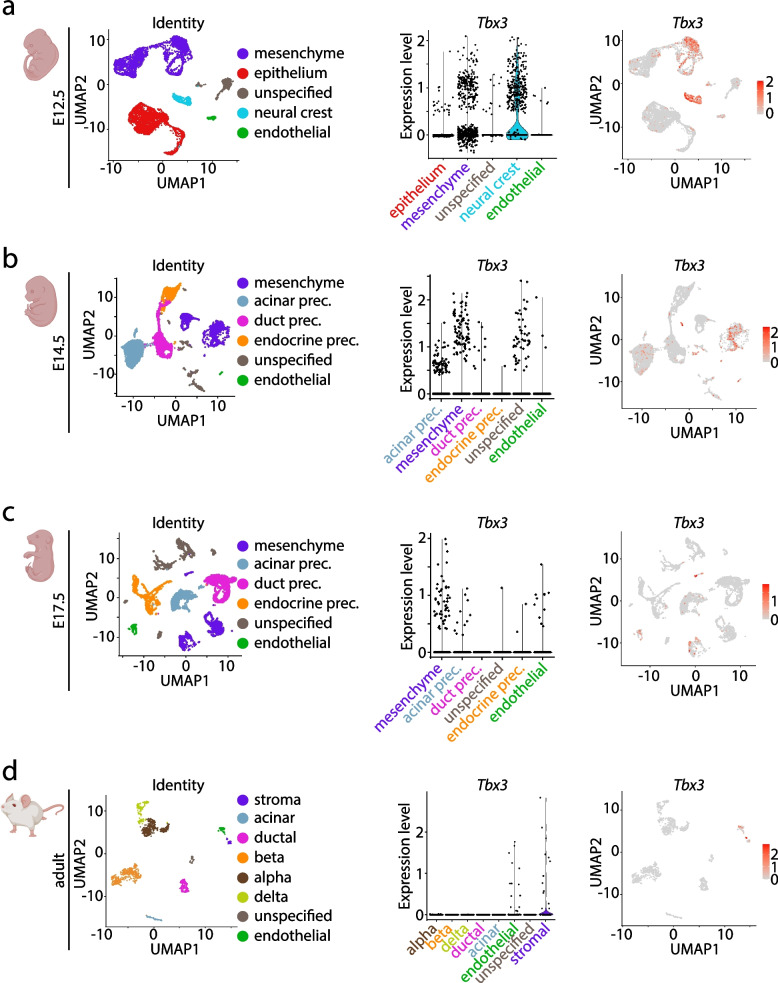
*Tbx3* expression during embryonic development of the pancreas. **a–d** Re-analysis of single-cell transcriptomic data sets [25, 26] from murine pancreata at indicated timepoints. UMAP cell-cluster representation of the re-analyzed single-cell RNA sequencing data (left panels) and expression patterns of *Tbx3* in embryonic pancreata as violin plots (middle panel) and featureplots (right panels) at **a** E12.5 (*n*=2 mice), **b** E14.5 (*n*=3 mice), **c** E17.5 (*n*=3 mice), and **d** in adult pancreata (*n*=7 mice). UMAP, uniform manifold approximation and projection. Expression levels depicts log-normalized counts

We first performed an RNA-based analysis to examine the differential expression patterns of *Tbx3* in mice by re-analyzing published datasets of mouse development (GSE101099) [25] as well as of adult murine pancreata (GSE109774) [26] revealing dynamic expression patterns over distinct stages (Fig. 1a–c). At E12.5 (embryonic day 12.5) and E14.5, *Tbx3* was expressed in various mesenchymal cells and neural crest cells, as indicated by cluster assignment using *Col3a1* or *Tlx2* [27, 28] (Fig. 1a,b, Additional file 1: Fig. S1a,b). Interestingly, while *Tbx3* expression levels were retained in a few cells of the mesenchymal compartment, only a limited number of endothelial cells expressed *Tbx3* at E17.5 as indicated by *Pecam1* positivity (Fig. 1c, Additional file 1: Fig. S1c). Of note, a few acinar precursor cells marked by *Cpa1* demonstrated expression of *Tbx3.* In the adult pancreas, *Tbx3* was detected only in few stellate (*Col3a1*) and endothelial (*Pecam1*) cells but not exocrine (*Cpa1*, *Krt19*) or endocrine (*Ins1*, *Gcg*, *Sst*) cells (Fig. 1d, Additional file 1: Fig. S1d).

To substantiate our analysis across another species, we investigated the expression of human *TBX3*. First, we chose to re-analyze our recently published bulk transcriptomic datasets (GSE131817) [29, 30] to assess *T-box* gene expression during in vitro differentiation of human PSCs toward pancreatic progenitor cells (Additional file 1: Fig. S2a,b). While other *T-box* genes (*TBX6*, *TBX19*, *EOMES*) were highly expressed in human pluripotent stem cells and in definitive endoderm, *TBX3* was among the most prominent transcripts at the pancreatic endoderm and pancreatic progenitor stage (Additional file 1: Fig. S2b). As mouse pancreatic progenitors start to arise and specify between E9.5 and E12.5 [31–35], this human analysis enabled us to zoom into earlier time points than those from the murine embryonic pancreata (Fig. 1a–c). To investigate *TBX3* expression in the more mature pancreatic lineages [36, 37], we investigated our recently published single-cell transcriptome dataset (GSE162547) monitoring, particularly pancreatic ductal differentiation [37]. Here, we noticed that *TBX3* expression was heterogeneously, still robustly expressed throughout the arising lineages, including ductal, endothelial, and endocrine cells, indicative of a widely distributed embryonic expression of *TBX3* during human development (Additional file 1: Fig. S2c). Next, we analyzed the time-resolved expression patterns of a set of *TBX3* interaction partners [38–40] in our published bulk transcriptomic dataset [29, 30] (Additional file 1: Fig. S2d). Here, we observed a decrease in well-known embryonic factors (e.g., *NANOG*), while several genes demonstrated higher expression levels over time at the pancreatic progenitor stage (e.g., *LEF1*).

To further elucidate the cell type-specific expression profiles of these *T-box* genes in adult pancreata, we performed a single-cell transcriptomic in silico analysis from four published human pancreatic islet donors (GSE84133) [41] (Additional file 1: Fig. S2e,f). In addition to endocrine cells (*GCG, INS; SST, PPY*), also exocrine cell types (marked by *KRT19* and *CPA1*), stellate (*COL3A1*), and endothelial (*PECAM1*) cells were identified (Additional file 1: Fig. S2f). As anticipated from the murine adult pancreata, the expression of *TBX3* was restricted to the stellate cells (Additional file 1: Fig. S2e).

Thus, *TBX3* is dynamically expressed during the differentiation of pancreatic cells at several developmental stages (PSC-derived pancreatic progenitors, duct-like cells, endothelial-like cells, and endocrine-like cells), and its expression shifts from epithelial cells to stromal cells in the adult human pancreas. Thus, expression in the adult human and murine organisms appears comparable in both species.

### TBX3 in site analysis confirms transcriptionally derived expression patterns

**Fig. 2:**
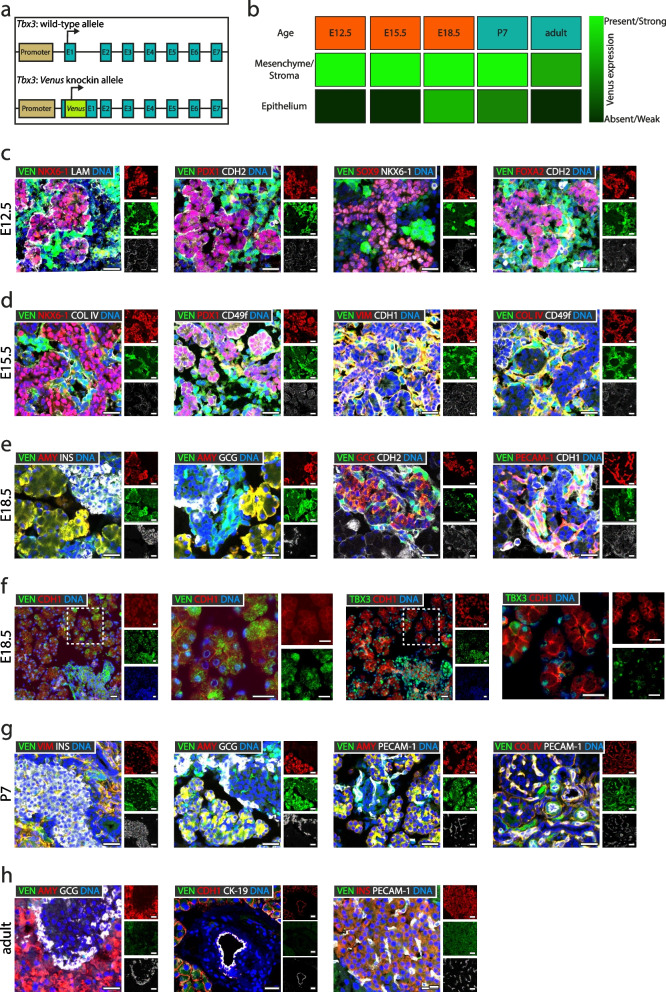
Venus reporter protein expression confirms RNA expression patterns of *Tbx3*. **a** Schematic representation of the *Tbx3*^*+/Venus*^ [42, 43] reporter mouse model employed to assess TBX3 expression patterns shown in **c–h**. Venus (VEN) expression is under the control of the *Tbx3* promoter and disrupts *Tbx3* expression from the knockin allele. **b** Schematic illustration depicting VEN expression pattern in pancreas during embryonic development and postnatal growth. **c** Immunofluorescence staining for NKX6-1/LAM (red/white), PDX1/CDH2 (red/white), SOX9/NKX6-1 (red/white), FOXA2/CDH2 (red/white), and Venus (VEN) (green) on E12.5 pancreata (*n*=3 mice). **d** Immunofluorescence staining for NKX6-1/COL IV (red/white), PDX1/CD49f (red/white), VIM/CDH1 (red/white), COL IV/CD49f (red/white), and VEN (green) on E15.5 pancreata (*n*=5 mice). **e** Immunofluorescence staining for pancreatic amylase (AMY)/INS (red/white), AMY/GCG (red/white), GCG/CDH2 (red/white), PECAM-1/CDH1 (red/white), and VEN (green) on E18.5 pancreata (*n*=4 mice). **f** Immunofluorescence staining for VEN (left panels) or TBX3 (right panels), both in green, in combination with CDH1 (red) on consecutive sections in E18.5 pancreata (*n*=1 mouse). **g** Immunofluorescence staining for VIM/INS (red/white), AMY/GCG (red/white), AMY/PECAM-1 (red/white), COL IV/PECAM-1 (red/white), and VEN (green) on early postnatal (P7) pancreata (*n*=4 mice). **h** Immunofluorescence staining for AMY/GCG (red/white), CDH1/CK-19 (red/white), INS/PECAM-1 (red/white), and VEN (green) on adult pancreata (*n*=3 mice). Cells were counterstained with DAPI. Scale bars correspond to 25 μm. *n*≥2 per group. E, exon

Dynamic *Tbx3* expression during pancreatic development prompted us to resolve stage-specific protein expression in the pancreatic anlage during embryonic and postnatal development. To visualize the activity of the murine *Tbx3* promoter at the protein level, we used a validated *Tbx3-Venus* (*Tbx3*^*tm1(Venus)Vmc*^) [11, 42–47] reporter system (Fig. 2a). In this model, the production of Venus protein reflects *Tbx3* promoter activity as detected with anti-GFP antibodies [42, 45]. By using a global knockout of *Tbx3* through the replacement of the first three exons by a *Cre*-knockin [48], we confirmed TBX3 antibody specificity (Additional file 1: Fig. S3a). Similarly, the anti-GFP antibody was licensed to capture the Venus protein in Tbx3-positive cells of the seminal vesicle serving as a positive control (Additional file 1: Fig. S3b). We chose multiple developmental stages (E12.5, E15.5, E18.5, adult) to visualize Venus expression. In addition, we investigated the pancreata of P7 (postpartum day 7 after birth) newborn mice. These experiments revealed a good correlation between protein and the scRNA-seq data (Fig. 1). Venus was significantly expressed in the pancreatic mesenchyme (Fig. 2b), a structure known to mediate inductive cues to pattern the pancreatic epithelium during organogenesis [49]. Specifically, at E12.5 Venus signal was detected around epithelial buds as indicated by co-staining for transcription factors NKX6-1, PDX1, SOX9, FOXA2, and basal membrane proteins such as laminin (LAM) (Fig. 2c). At E15.5, the expression pattern of Venus was virtually the same as at E12.5, demonstrated by colocalization with mesenchymal markers vimentin and collagen IV (COL IV), but not the epithelial markers NKX6-1, PDX1, CDH1, and CD49f (Fig. 2d). Of note, Venus protein was detected both in the mesenchymal and epithelial compartment of the pancreas at E18.5 and P7 whereas RNA expression of *Tbx3* was more abundant in mesenchymal and endothelial cells at E17.5 (Figs. 1d and 2e-g). However, concordant to the few acinar precursor cells expressing *Tbx3* at E17.5 (Fig. 1c), the epithelial expression was mostly in acinar cells as proven by colocalization of Venus with amylase (AMY) and not in endocrine cells labeled by insulin (INS) and glucagon (GCG). Furthermore, Venus was detected in mesenchymal cells (collagen IV +), and also detected in PECAM-1 expressing endothelial cells. To further validate the expression of Venus in acinar (precursor) cells at E18.5, we performed staining of Venus and TBX3 on consecutive sections of E18.5 pancreata (Fig. 2f) confirming the expression of both markers in acinar structures (Zoom-ins in Fig. 2f). Immunohistochemistry analysis further licensed the acinar expression pattern (Additional file 1: Fig. S3c). In adult mice, concordant with the RNA data, Venus was not detected in pancreas-specific epithelial (CDH-1, AMY, CK-19, INS, GCG) cell types (Figs. 1e and 2h). Thus, TBX3 protein expression, as assayed with the Venus reporter, presents specific and developmentally dynamic expression patterns in both the pancreatic epithelium and the mesenchyme. These patterns correlate mostly with the RNA findings, except the weak but robust acinar expression around E18.5 which appears underestimated in scRNA sequencing data potentially attributed to technical limitations for weakly expressed genes [50–54].

### Compartment-specific deletion of Tbx3 leads to virtually unaltered pancreatic development

**Fig. 3:**
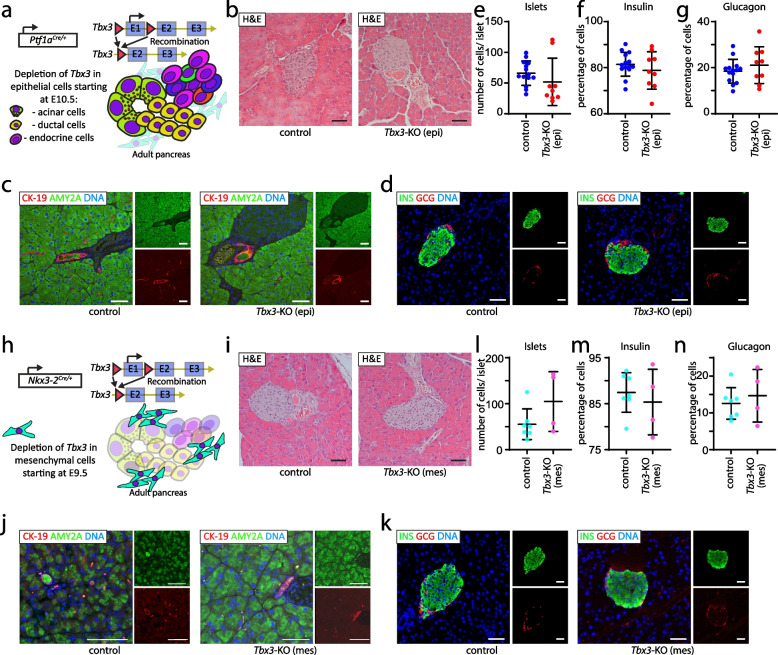
*Tbx3* is largely dispensable for pancreatic development. **a** Schematic representation of the *Tbx3*^*fl/fl*^*; Ptf1a*^*Cre*/+^ mouse model allowing *Tbx3* deletion in pancreatic epithelial tissue (*Tbx3-*KO (epi)). **b** Hematoxylin and eosin (H&E)-stained histological sections of control and *Tbx3-*KO (epi) pancreata. Immunofluorescence stainings for **c** CK-19/AMY2A (red/green) and **d** INS/GCG (green/red) on control and *Tbx3-*KO (epi) pancreata. Quantification of **e** number of cells per islet based on INS/GCG co-stainings, **f** percentage of insulin expressing cells in islets, and **g** percentage of glucagon-expressing cells in Langerhans islets. *n*=14 control (littermates of *Tbx3-*KO (epi)) pancreata and *n*=9 *Ptf1a-Cre*-driven *Tbx3-*KO (epi) pancreata. **h** Schematic representation depicting *Tbx3*^*fl/fl*^*; Nkx3-2*^*Cre/+*^ mouse model allowing *Tbx3* depletion in pancreatic mesenchyme (*Tbx3-KO* (mes)). **i** H&E-stained histological sections of control (littermates of *Tbx3-*KO (mes)) and *Nkx3-2-Cre*-driven *Tbx3-KO* (mes) pancreata. Immunofluorescence stainings for **j** CK-19/AMY2A (red/green) and **k** INS/GCG (green/red) on control and *Tbx3-KO* (mes) pancreata. Quantification of **l** number of cells per islet area based on INS/GCG co-stainings, **m** percentage of insulin expressing cells in islets, and **g** percentage of glucagon-expressing cells in Langerhans islets. *n*=7 control (littermates of *Tbx3-*KO (mes)) pancreata and *n*=4 *Nkx3-2-Cre*-driven *Tbx3-*KO (mes) pancreata. Cells were counterstained with DAPI. Scale bars correspond to 50 μm. Data are expressed as individual datapoints and mean ± SEM. Mann-Whitney test was performed to investigate significance levels (no significance reached)

To study the functional role of TBX3 in the context of pancreatic organogenesis, two different conditional knockout models targeting *Tbx3* in either the pancreatic epithelium or mesenchyme were employed. Embryonic deletion of *Tbx3* in the epithelial compartment of the pancreas was achieved by crossing *Tbx3*^*flox/+*^ with *Ptf1a*^Cre/+^ mice, a model known to target all epithelial lineages of the pancreas, including acinar, ductal, and most endocrine cells starting from E10.5 (Fig. 3a) [35]. Specific recombination in epithelial cells of the pancreas was confirmed by immunohistochemical stainings for tdRFP in *Ptf1a*^Cre/+ ^ [35] x *LSL-tdRFP*^*KI/KI*^ [55, 56] mice (Additional file 1: Fig. S3d) [56]. Histologic morphology was similar between homozygous knockout mice (*Tbx3*-KO (epi, epithelial)) and control mice (no knockout of *Tbx3*) (Fig. 3b). Furthermore, immunofluorescence analysis labeling exocrine pancreatic markers (CK-19, AMY2A; Fig. 3c) revealed virtually no difference between *Tbx3*-KO (epi) and control mice. This was also valid for the endocrine compartment, where only subtle differences with a trend toward smaller islets in *Tbx3*-KO (epi) mice was documented while maintaining similar percentages of INS+ beta-cells and GCG+ alpha-cells (Fig. 3d–g).

Given the essential role of the pancreatic mesenchyme for proper lineage formation [49, 57], *Tbx3* was also deleted in this compartment. The *Nkx3-2*^Cre/+^ mouse strain was used (Fig. 3h) as it has been employed to demonstrate the relevance of the pancreatic mesenchyme for proper pancreatic development [49]. As with the epithelial compartment, homozygous deletion of *Tbx3* (*Tbx3*-KO (mes, mesenchymal)) in the mesenchyme did not alter pancreatic morphology (assayed by histomorphologic investigation of H&E staining) (Fig. 3i). Additional analysis of exocrine markers (CK-19 and AMY2A) showed no difference between *Tbx3*-KO (mes) and control mice (Fig. 3j). Interestingly, also the investigation of the endocrine markers insulin and glucagon revealed no significant difference (Fig. 3k–n). The size of islets (Fig. 3l) and the percentages of insulin- and glucagon-expressing cells (Fig. 3m,n) were not significantly different between *Tbx3*-KO (mes) and control mice. Thus, although *Tbx3* displayed specific and dynamic regulation patterns on mRNA and protein levels in both mesenchyme and epithelium, its targeted ablation in either compartment does not relevantly alter proper pancreatic organ formation.

To check whether the well-described homolog TBX2 might compensate for TBX3 loss [9, 10], we performed immunohistochemistry for TBX2 in pancreata of both knockout models (Additional file 1: Fig. S4a,b). However, the overall staining intensity remained similar in control (epi) and *Tbx3*-KO (epi), as well as control (mes) and *Tbx3*-KO (mes) pancreata, albeit nuclear and cytoplasmatic protein distribution varied across various animals without a relevant genotype-specific trend.

Finally, we wanted to challenge the relevance of *TBX3* in human pancreatic development. Thus, we employed an inducible knockdown of *TBX3* by an shRNA in a previously reported iPSC line [12] during in vitro pancreatic progenitor differentiation [58–61]. We assessed trilineage potential (generation of acinar, ductal, and endocrine cells) in a recently described porcine urinary bladder (PUB) organ culture model for the maturation of pancreatic progenitor cells [62, 63] (Additional file 1: Fig. S5a). Of note, we did observe the generation of morphologically similar (Additional file 1: Fig. S5b), ductal (Additional file 1: Fig. S5c), acinar (Additional file 1: Fig. S5d), and endocrine (Additional file 1: Fig. S5e) structures on the PUB scaffold. Thus, the trilineage potential was not significantly altered by *TBX3*-knockdown during pancreatic progenitor differentiation. We conclude that *TBX3* is dispensable for proper pancreatic organ formation in mice and men.

### Loss of Tbx3 results in increased fibrosis and excessive proliferation during recovery from acute pancreatitis

**Fig. 4:**
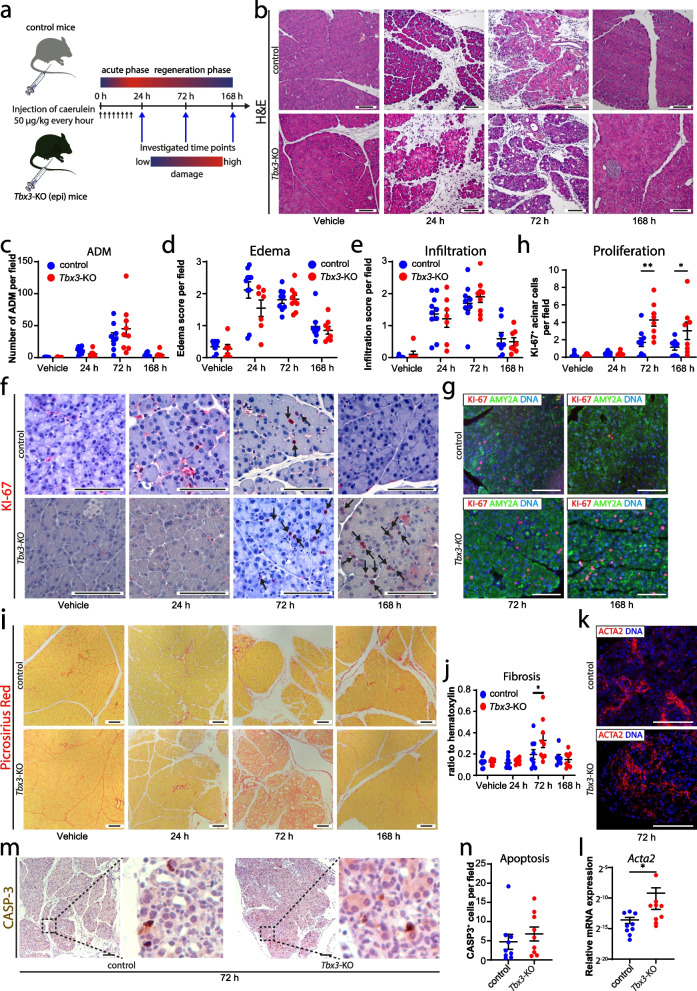
*Tbx3* loss leads to overshoot proliferation of acinar cells and accumulation of fibrosis. **a** Schematic representation of the caerulein-induced acute pancreatitis assay shown in **b–l**. Mice were euthanized at the indicated time points (arrows in blue). **b** Hematoxylin and eosin (H&E)-stained histological sections of control littermates of *Tbx3-*KO (epi) and *Tbx3*^*fl/fl*^*; Ptf1a*^*Cre*/+^ (*Tbx3-*KO (epi)) pancreata after treatment with caerulein or vehicle. **c** Quantification of acinar-to-ductal metaplasia (ADM), **d** edema score, **f** inflammatory infiltration score per field in pancreata from caerulein-induced acute pancreatitis assay shown in **a** (*n*≥6 per group). **f** Representative immunohistochemistry stainings for KI-67 and **g** immunofluorescence co-staining of KI-67/AMY2A (red/green) at indicated timepoints. **h** Quantification of KI-67-positive acinar cells per field in pancreata from caerulein-induced acute pancreatitis assay shown in **a** (*n*≥6 per group, detailed in Additional file 3). **i** Picrosirius red stained histological sections of pancreata from caerulein-induced acute pancreatitis assay shown in **a**. **j** Quantification of picrosirius red-positive area as ratio to hematoxylin-based whole pancreas surface in pancreata from caerulein-induced acute pancreatitis assay shown in **a** (*n*≥6 per group). **k** Immunofluorescence staining for ACTA2 (red) in pancreata at 72h after induction of the acute pancreatitis. **l** Relative mRNA expression of *Acta2* at 72h after induction of acute pancreatitis. **m** Representative immunohistochemistry staining for caspase-3 (CASP3) at the 72h timepoint and **n** respective quantifications. Cells were counterstained with DAPI for immunofluorescence analysis. Graphs present individual data points and mean with SEM. Two-way ANOVA with Sidak’s post-test was performed for graphs with multiple time points. Mann-Whitney test was performed to assess significance at single time points for apoptosis and *Acta2*. *, *p* < 0.05; **, *p* < 0.01. Scale bars represent 100 μm

Since pancreatic development shares common regulatory patterns with pancreatic repair, we challenged *Tbx3*-KO (epi) mice and control mice with caerulein-induced acute pancreatitis to investigate the potential relevance of *Tbx3* under exogenous stress conditions (Fig. 4a). Notably, no significant differences between control and *Tbx3*-KO mice were detected based on the amount of acinar-to-ductal metaplasia (ADM) as well as edema and inflammatory infiltration scores (Fig. 4b–e). As expected, a substantial increase of ADMs at 72h preceded by increased edema and infiltration of inflammatory cells (24h) was documented after induction of caerulein-driven acute pancreatitis in both groups of animals. The damage was largely repaired 7 days after the first injection. However, when assessing the proliferation rates of acinar cells with normal morphological configuration by KI-67 quantification, we noticed a significantly higher amount of KI-67^+^ cells per field in the *Tbx3*-KO (epi) mice compared to the control counterpart at 72h (4.3 cells/per field vs 1.7 cells/field) and 168h (3.0 cells/field vs 1.2 cells/field) (Fig. 4f–h). Interestingly, increased acinar proliferation was accompanied by significantly more fibrosis as quantified upon Sirius Red stainings at 72h in *Tbx3*-KO (epi) mice (Fig. 4i,j). The amount of fibrosis was reflected by the presence of ACTA2-positive cells around ADM structures (Fig. 4k). Interestingly, gene expression analysis of *Acta2* at 72h after the onset of pancreatitis confirmed the significant difference between *Tbx3*-KO (epi) and control mice (Fig. 4l). Finally, we checked if apoptosis at 72h after induction of pancreatitis might play a role in the altered regeneration (Fig. 4m,n). However, no significant differences were observed concerning the rate of apoptotic cells. Thus, despite the fact that regeneration was achieved in *Tbx3*-KO (epi) mice, prolonged proliferation in acinar cells and accumulation of fibrosis suggest that *Tbx3* is involved in proper regeneration from organ injury, but its absence is well compensated by other genes.

### Loss of Tbx3 results in increased susceptibility of acinar cells to fibroinflammatory stimuli

**Fig. 5:**
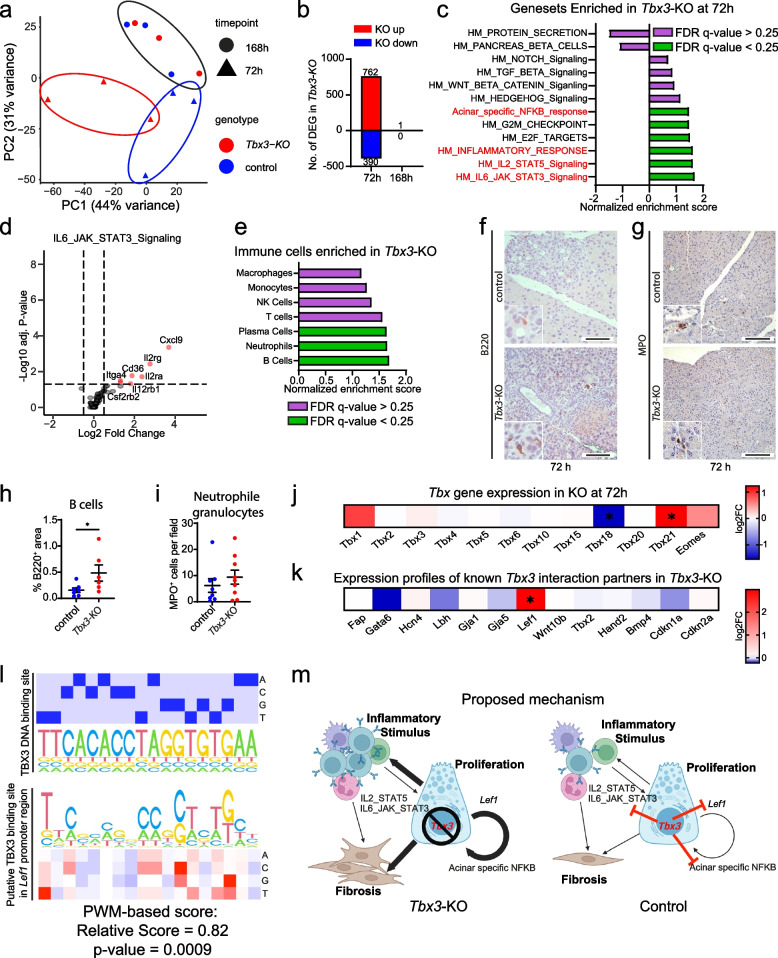
Whole transcriptome analysis uncovers a potential role of *Tbx3* to fine-tune fibroinflammatory stimuli. **a** Principal component analysis of *n*=3 mice per genotype (control littermates of *Tbx3-*KO (epi) and *Tbx3*^*fl/fl*^*; Ptf1a*^*Cre*/+^ (*Tbx3-*KO (epi))) at 72 and 168h after induction of acute pancreatitis. **b** Differentially expressed genes (DEG) in *Tbx3-*KO (epi) mice at indicated timepoints. **c** Gene set enrichment analysis (GSEA) in *Tbx3-*KO (epi) of different hallmark (HM) gene sets and one acinar-specific upregulated gene set in response to NF-KB (Supplementary Table 1) [64] at 72h. **d** Volcano plot of the enriched IL-6-JAK-STAT3-Signaling in *Tbx3-*KO (epi) mice at 72h. **e** GSEA of immune cell signatures [65] in *Tbx3-*KO (epi) mice at 72h. **f** Immunohistochemistry staining for B220+ B cells at 72h after induction of acute pancreatitis . **g** Immunohistochemistry staining for MPO+ neutrophils at 72h after induction of acute pancreatitis. **h** Quantification of percentages of B220+ B cell area per field of view in *n*=7 control (littermates of *Tbx3-*KO (epi)) pancreata and *n*=6 *Ptf1a-Cre*-driven *Tbx3-*KO (epi) pancreata. **i** Quantification of MPO+ neutrophils per field of view in *n*=8 control (littermates of *Tbx3-*KO (epi)) pancreata and *n*=9 *Ptf1a-Cre*-driven *Tbx3-*KO (epi) pancreata. Graphs present individual data points and mean with SEM. Mann-Whitney test was performed to assess significance. *, *p* < 0.05. **j** Heatmap of log2 fold changes of *Tbx* genes at 72h in *Tbx3-*KO (epi) pancreata compared to control pancreata. **k** Heatmap of log2 fold changes of known interaction partners of *Tbx3* at 72h in *Tbx3-*KO (epi) pancreata compared to control pancreata. **l** DNA footprint analysis of putative DNA binding sites in the promoter of *Lef1* at −18bp from TSS. PWM-based score 0.82, *p*-value=0.0009. **m** Proposed mechanism for *Tbx3*-related fine-tuning of acute pancreatitis. *, adjusted *p* < 0.05. Scale bars represent 100 μm. MPO, myeloperoxidase.

To further specify the subtle differences across the two genotypes during regeneration (*Tbx3*-KO (epi) vs. control (epi) mice), we performed RNA sequencing for the two most interesting time points (72 and 168h). Interestingly, we noticed a clear difference in the principal component analysis (PCA) of whole transcriptomes from *Tbx3*-KO (epi) mice compared to their respective controls at 72h, while the differences at 168h were virtually absent (Fig. 5a). We could identify 1152 differentially expressed genes (DEG) at 72h (762 up, 390 down in *Tbx3*-KO (epi)), while only 1 DEG was detected at 168h (Fig. 5b). Subsequently, we performed gene set enrichment analysis (GSEA) for hallmark (HM) gene sets and one acinar-specific NF-κB response gene set (Additional file 2: Supplementary Table 1) adapted from [64] (Fig. 5c). Interestingly, the significantly enriched gene sets included the Il6-Jak-Stat3-, Il2-Stat5-signaling, Inflammatory-Response, E2F-Targets, G2M-Checkpoint, and the acinar-specific NF-κB response. Notably, most gene sets are related to the immune system. We also checked other well-known gene sets relevant to pancreatic organ regeneration and detected no significant enrichment for the *HM-Hedgehog, Wnt, Tgf-β,* and *Notch signaling*. Concerning depleted gene sets, we noticed a non-significant depletion of *Pancreatic-Beta-Cell-signature* and *Protein-Secretion-Signature*, the latter being potentially indicative of normal acinar function.

Fitting with the enrichment of the Il6-Jak-Stat3 gene signature, a variety of immune cell-related genes (e.g., *Cxcl9*, *Il2rg*) were significantly overexpressed at 72h post pancreatitis (Fig. 5d), which prompted us to investigate specific immune cell signatures (Additional file 2: Supplementary Table 2 [65]; (Fig. 5e). Interestingly, signatures for B cells and neutrophils were significantly upregulated. However, validation on protein level could only confirm an increased abundance of B cells in *Tbx3*-KO (epi) pancreata as compared to control (epi) counterparts at 72h after induction of pancreatitis (Fig. 5f,h). Neutrophil granulocyte quantification revealed only a trend (Fig. 5g,i). Infiltrating T cells and macrophages remained similar across the two genotypes (Additional file 1: Fig. S6a-d). To identify potential compensators for *Tbx3* loss in the epithelial compartment, we analyzed the expression of all expressed *Tbx* genes at 72h (Fig. 5j). Interestingly, most *Tbx* genes remained unaltered, with *Tbx18* being significantly downregulated and *Tbx21* overexpressed in the *Tbx3*-KO (epi) mice. Next, a set of Tbx3 target genes were investigated [38] (Fig. 5k). Amongst these, the sole strongly and significantly upregulated gene was *Lef1*. Accordingly, we calculated the putative binding score and significance using the palindromic TBX3 binding site (18 bp) for the *Lef1* promoter consensus sequence. Indeed, the relative similarity score was highly significant (score = 0.82, *p* = 0.0009), indicating direct TBX3 binding to the *Lef1* promoter, most likely triggering its transcriptional repression (Fig. 5l).

## Discussion

In this study, we (i) determined the expression of *TBX3* during pancreatic organogenesis, (ii) demonstrated expression switching between mesenchymal and epithelial cells during pancreatic development, and (iii) revealed expression in the stromal compartment of the adult pancreas. Surprisingly, epithelial and mesenchymal knockout of *Tbx3* did not lead to statistically significant phenotypic alterations in the murine pancreas, and knockdown of *TBX3* in a human iPSC-based system for pancreatic differentiation did not alter ex vivo pancreatic development. It is likely that *TBX3* function may be compensated by other genes in these compartments during development. Driven by the fact that developmental programs can become re-activated during regeneration from injury, we investigated the relevance of *Tbx3* for regeneration after caerulein-induced acute pancreatitis. Our findings indicate that epithelial deletion of *Tbx3* does not significantly impact overall organ regeneration but does prolong proliferation and increase fibrosis. Again, this lack of a major effect in the face of *Tbx3* ablation is likely attributable to other factors, while *Tbx3* in itself is required for the fine-tuning of the regenerative process. Whole transcriptome analysis finally revealed putative mechanisms that alter the regeneration via enhanced immune cell-acinar interactions, fibroinflammatory stimuli, and dysregulated proliferation (Fig. 5m). Altogether, while *Tbx3* is not a master regulator of pancreatic development and organ regeneration, our data show a relevant but subtle contribution of this factor to adult pancreatic organ homeostasis and development.

Interestingly, *Tbx3* RNA expression levels demonstrated a virtually lower percentage of *Tbx3+* cells than assayed in immunofluorescence analysis. This discrepancy was higher for acinar cells than for mesenchymal or stromal cells, which may be related to either technical limitations of single-cell RNA-seq [51–54] with a bias to filter out genes with low expression [50] and eventually boosted by the fact that the protease- and ribonuclease-rich acinar cells raise difficulties to isolate intact RNA [66–68]. Another potential explanation would be that *Tbx3* expression levels in mesenchymal/stromal cells are, in general, higher (also evident in the violin plots of Fig. 1), which eases the detection.

Nevertheless by employing a knockout-validated antibody and a Venus reporter system [11, 42–47], the expression of TBX3, or Venus, respectively, was proven on the protein level. In addition, the evidence of human *TBX3* expression in an in vitro iPSC differentiation in the epithelial compartment highlights that *TBX3* is indeed expressed during embryonic pancreatic development.

Indeed, important roles for TBX3 have been shown in lung branching morphogenesis and ureter organogenesis [9, 10]. In both cases, TBX3 was located in the mesenchyme, and depletion of *Tbx3* (together with *Tbx2*, which otherwise would have compensated *Tbx3*) resulted in defective organ patterning [9, 10]. Based on such recent publications, we chose to not only perform a knockout of *Tbx3* in the epithelial compartment by *Ptf1a-Cre* but also in the mesenchymal compartment by *Nkx3-2-Cre.* Other than our anticipation from the expression level profiles and the recent publications [9, 10], neither of the knockout models led to a strong phenotype. *TBX2* and *3* are members of the same T-box subfamily [1]. Their partially redundant roles in lung or ureter development [5, 9, 10] raise the question if this leads to redundant function in pancreatic evolution. However, our solely on gene and protein quantification relying study was underpowered to detect evidence for TBX2 compensating during pancreatic development as TBX2 remained unchanged in both the two knockout models and the pancreatic injury model. To ultimately clarify this hypothesis, a double-knockout of *Tbx2* and *Tbx3* in both pancreatic compartments would be necessary. However, this was clearly beyond the scope of the current study.

Even though we did not observe a gross impact of *Tbx3* on recovery from acute pancreatitis, we observed (i) higher amounts of fibrosis, (ii) prolonged proliferation in acinar cells in its absence, and (iii) higher variance in some investigated parameters of regeneration of *Tbx3*-KO mice. Of note, we assessed the putative compensation by other *Tbx* genes during pancreatitis. Here we observed an upregulation of *Tbx21.* However, as *Tbx21* (*T-bet*) and also *Eomes* (increased expression, though not significant) also play relevant roles in immune cell signaling, we attributed the enhanced expression to the increased inflammatory signature. Considering the downregulation of *Tbx18*, we pinpoint a recent publication of cardiac pacemaker differentiation where *Tbx3* and *Tbx18* are co-regulated, implying that a similar program could occur during pancreatic regeneration [69].

Based on the fact that *Tbx3* is involved in the regulation of Wnt signaling in embryonic development [9] and Wnt signaling controls the proliferation of acinar cells during organ regeneration [24], *Tbx3* may have a regulatory role on Wnt signaling during acinar cell regeneration. By demonstrating that *Lef1*, a central mediator of the Wnt signaling axis [24, 70–73], is not only significantly upregulated in *Tbx3*-KO (epi) mice but also possesses a putative DNA binding site for TBX3 in its promoter region, we postulate a previously unknown potential suppressive function of TBX3 on *Lef1* building the following hypothetical model: The absence of *Tbx3* in the pancreatic epithelium could trigger excessive proliferation eventually involving *Lef1*, a known member of the Wnt signaling [74, 75], and acinar-specific upregulated NF-KB-pathway members leading to abnormal proliferation of acinar cells [64]. Such proliferation pulse is accompanied by an increased fibroinflammatory response resulting in transient accumulation of B cell-infiltrated fibrotic tissue (Fig. 5m). This suggests an ongoing homeostatic process during pancreatic regeneration from an injury involving only *Tbx3* for fine-tuning without the generation of substantial alterations upon genetic loss.

## Conclusions

In summary, *Tbx3* shows switching expression patterns in the developing pancreas. Although *Tbx3* seems dispensable for proper pancreatic development and lineage entry, its absence results in altered organ regeneration after induction of acute pancreatitis marked by enhanced fibrosis and inflammation.

## Methods

### Single-cell RNA sequencing re-analysis

Expression patterns of *Tbx3* on a single-cell level were derived from two different murine datasets: GSE101099 [25] for embryonic pancreata at E12.5, E14.5, and E17.5, as well as GSE109774 for adult pancreata [26]. Expression of *TBX3* in a recently published human pancreatic duct-like cell differentiation approach from pluripotent stem cells was performed as recently described in detail (GSE162547) [37]. The expression of *TBX3* in adult pancreata of four human donors was interrogated in a published dataset: GSE84133 [41]. Before expression analysis, preprocessing steps were conducted in RStudio with the version 4.0.4 and the “dplyr”, “Seurat” version 4.0.6 [76–79], and “patchwork” packages. For the murine datasets, cells with less than 1000 expressed genes, more than 4000 genes for E12.5, E14.5, and more than 5000 genes for E17.5 and genes expressed in fewer than 3 cells were filtered out before analysis. For GSE109774, cells expressing more than 5% ERCC RNA spike-ins were additionally removed. For human datasets, cells expressing either less than 300 genes or more than 4000 genes and genes expressed in fewer than 10 cells were filtered out. Data were merged for E12.5 for GSM2699156_E12_B2 and GSM3140915_E12_v2, for E14.5 for GSM2699154_E14_B1, GSM2699155_E14_B2, and GSM3140916_E14_v2, and for E17.5 for GSM2699157_E17_B2, GSM3140917_E17_1_v2, and GSM3140918_E17_2_v2 of the respective dataset. Human datasets were also merged. Batch effects were corrected with the FindIntegrationAnchor() and the IntegrateData() function in the reciprocal PCA approach of the Seurat workflow. Datasets were Log-normalized, and the top 2000 highly variables genes were identified by the “vst” method for the murine datasets. For the human datasets, similar to our recent work [59], the top 4000 highly variable genes were identified. Data were scaled. A standard Seurat workflow was performed. Single-cell neighborhood was calculated with the first 30 principal components and clustering was performed with the Louvain algorithm with a resolution of 0.015 for E12.5, 0.5 for E14.5, 0.5 for E17.5, 0.2 for the adult murine pancreas, and 0.5 for human datasets. Cluster identity was annotated by specific marker genes.

### Bulk RNA expression re-analysis from publicly available RNA sequencing data sets

RNA expression levels of different *T-Box* transcription factors in embryonic stem cells, definitive endoderm, pancreatic endoderm, and pancreatic progenitors were derived as FPKM (fragments per kilobase of transcript per million mapped reads) from a previously published dataset (GSE131817, wildtype samples) [29, 30]. Data were analyzed in RStudio with the R version 4.0.4. The heatmap was generated with the “pheatmap” package (version 1.0.12), scaling was set to columns, and clustering followed the “ward.D2” method. A list of described *TBX3* interaction partners was derived from [38]. A heatmap was generated as indicated above from the same dataset (GSE131817) [29, 30]. The scale was set to the row factor.

### Ethics statement

All animal care and procedure were conducted in compliance with the German legal regulations and were previously approved by the local governmental review board of the state of Baden-Württemberg (Permission no. 1477, O.195-4, O.195-6, O.195-10, and O.195-12) or conducted in compliance with the local government of Bavaria. All mouse work aspects were performed according to acknowledged guidelines of the Society of Laboratory Animals (GV-SOLAS) and of the Federation of Laboratory Animal Science Associations (FELASA).

### Mice

*Ptf1a*^*Cre*^ (*Ptf1a*^*tm1(cre)Cvw*^) mouse strain [35] was previously described [80, 81]. *Nkx3-2*^*Cre*^ (*Nkx3-2*^*tm1(cre)Wez*^) was a kind gift of Warren E. Zimmer (Texas A&M University) [82]. *Tbx3*^*flox*^ (*Tbx3*^*tm3.1Moon*^) (kind gift of Anne M. Moon) [4], and *Tbx3*^*Venus*^ (*Tbx3*^*tm1(Venus)Vmc*^) mouse (kind gift of Vincent M. Christoffels) [42] strain were maintained on a complex C57BL/6×129/Sv genetic background. Mice were housed and bred in a conventional health status-controlled animal facility. All animal care and procedures followed German legal regulations and if applicable were previously approved by the governmental review board of the state of Baden-Württemberg. All the aspects of the mouse work were carried out following strict guidelines to insure careful, consistent, and ethical handling of mice.

### Analysis of Ptf1a^Cre^-specific recombination in adult pancreata

Formalin-fixed paraffin-embedded (FFPE) tissue sections of pancreata of *Ptf1a*^*Cre*^ [35] x *R26-LSL-tdRFP*^*KI*^ [55] mice which were previously described [56] were a kind gift of Patrick Hermann (Ulm University Hospital, Department of Internal Medicine I).

### Knockout validation of TBX3-antibodies

TBX3 antibody was analyzed by immunofluorescence in archived *Tbx3*^*Cre/Cre*^ at E13.5 or *Tbx3*^*wt/wt.*^ [48].

### Mouse embryos and adult pancreata preparation

Embryos were collected at 12.5, 15.5, and 18.5 days post-coitum. Newborn mice were euthanized at post natal day 7 (P7). Pancreata were fixed in 4% PFA for 2 h at room temperature or for 16 h at 4°C, cryopreserved, and embedded in optimal cutting temperature compound (OCT compound). Adult mice were at least 8 weeks old. Pancreata were fixed in cold 4% PFA for 16 h at 4°C and embedded in paraffin for histological analysis.

### Model of Caerulein-induced acute pancreatitis

Acute pancreatitis in *Tbx3*-KO (epi) and control mice was induced in adult animal (≥ 8 weeks) by hourly injections (8 times) of 50 μg caerulein /kg bodyweight (Sigma-Aldrich) dissolved in PBS (vehicle). Mice were sacrificed 24, 72, and 168 h after the first caerulein injection. Corresponding volumes of vehicle were injected in *Tbx3*-KO (epi) and control mice, and pancreata were isolated 24 h after the first injection.

### Differentiation of pancreatic progenitor cells and organ culture model

Maintenance culture and differentiation to pancreatic progenitors of a well-described iPSC line with a doxycycline-inducible shRNA against *TBX3* [12] was performed as described recently in a step-by-step protocol [58, 59] with slight modifications. For shRNA expression, 3 μg/mL doxycycline was added to the cells. The medium was changed on a daily basis. After the differentiation of pancreatic progenitor cells, organ culture of porcine urinary bladders was performed as described recently [62, 63]. Briefly, porcine urinary bladders (PUB) were cleaned, de-epithelialized, and sterilized with 0.1% peroxy-acetic acid. In total, 500,000 cells per ring were seeded in 30 μL of 50% Matrigel and 50% basal medium as in [58, 59], including 5% FCS and 10 μM Y-27632. The medium of PUBs was changed once a week, and doxycycline was added twice a week freshly. After 2 weeks, PUBs were fixed in 3.7% formaldehyde and processed for histology as described recently [62, 63].

### Histology

All histological experiments on FFPE tissue were performed as previously described [20, 59, 62]) following standard procedures. Four-micrometer-thin sections were rehydrated. Heat-mediated antigen retrieval was either performed with citrate-based buffer (pH=6) or TRIS-based buffer (pH=9). Blocking was performed in 5% normal donkey serum in 0.1% Triton X-100 in PBS for 30 min at RT. Primary and secondary antibodies and respective dilution factors are listed in Additional file 2: Supplementary Table 3. Bright-field images were acquired using a Leica DM5500B microscope (Leica) equipped with a Leica DMC5400 camera and Leica Application Suite software (Leica) or by using a Zeiss Axioscope2 microscope (Carl Zeiss) ZEN3.1 imaging software (Carl Zeiss). Immunofluorescence images were obtained with a Zeiss Axioscope2 microscope equipped with an Axiocam 702 (Carl Zeiss). Acquired pictures were subsequently analyzed using ImageJ software (National Institutes of Health).

Immunofluorescence stainings of cryosections were performed following standard protocols. Briefly, 10-μm-thick cryo-sectioned pancreata were rehydrated in PBS for 30 min, washed twice with PBS containing 0.1 % Tween 20 (PBS-T), and permeabilized using 0.1 M glycine (Merck) and 0.1% Triton X-100 (for NKX6.1: 0.5% Triton X-100; Merck) in MilliQ water for 15 min at RT and blocked using 0.1% Tween - 20, 10% heat inactivated fetal calf serum (FCS), 0.1% BSA, and 3% donkey serum in PBS for 1 h at RT. Incubation with primary antibodies diluted in blocking solution occurred overnight at 4°C or 1 h at RT. The slides were washed 3× with PBS-T for 10 min each, and subsequently, secondary antibody solution was added for 2 h at RT. DAPI/PBS solution was added for 20 min before washing of slides 3× with PBS for 10 min each. Finally, the slides were mounted with Elvanol and kept 24 h at RT to dry.

### Histological quantifications

The total number of cells per Langerhans islets, as well as the number of insulin and glucagon-expressing cells, was counted manually from at least 3 different islets per pancreas on one section. Acinar-to-ductal metaplasias (ADMs) were quantified by counting at least ten 200× fields. Edema and immune cell infiltration were scored from 0 to 3 (0, no evident edema to 3, maximal degree of edema; 0, no immune infiltration to 3 maximal degree of immune infiltration) as previously described [20]. Proliferation was quantified by counting KI-67-positive cells in immunohistochemistry from ten 400× fields of each pancreas. KI67-positive cells within only clearly identifiable acinar structures were considered. Immune cells were excluded from the quantification. For picrosirius red-positive area/hematoxylin-based whole pancreas surface ratio quantification, pictures from at least five 100× fields were loaded into ImageJ software (National Institutes of Health) to perform color deconvolution. Areas covered by picrosirus red (red) and hematoxylin (yellow) were quantified automatically. Immune cell populations were quantified by counting the absolute number of positive cells per field in at least ten 200× fields. Caspase-3 (CASP3)-positive cells per field of view were quantified manually by counting the number of positive cells in each pancreas in at least six 100× fields. Monocytes, neutrophils, and T cells were manually quantified in at least five different 200× fields. B cells were quantified in at least 8 different 200× fields by quantifying the B220 positive area per field in ImageJ.

### RNA extraction, cDNA synthesis, qRT-PCR

RNA extraction was performed as described previously [20] with the RNeasy Plus Mini Kit (Qiagen) according to the manufacturer’s instructions. RNA concentration was determined with a NanoDrop. cDNA synthesis was performed with the iScript™ cDNA Synthesis Kit (Bio-Rad) following the manufacturer’s instruction. Briefly, 1 μg of RNA was transcribed. cDNA was diluted 15-fold. PCR was performed at the Rotor-Gene-Q (Qiagen) using 4 μL of diluted cDNA with 5 μL of Green Master Mix (Genaxxon) and 0.4 μM forward and reverse primer (each 0.5μL). Gene expression was normalized to ribosomal protein *S18*. Primer (Biomers) sequences are as follows: *Acta2*: fwd 5′-GTTCAGTGGTGCCTCTGTCA-3′, rev 5′-ACTGGGACGACATGGAAAAG-3′, *S18*: fwd 5′-GTAACCCGTTGAACCCCATT-3′, rev 5′- CCATCCAATCGGTAGTAGCG-3′.

### RNA sequencing

The amount of total RNA was quantified using the Qubit 2.0 Fluorometric Quantitation system (Thermo Fisher Scientific, Waltham, MA, USA), and the RNA integrity number (RIN) was determined using the Experion Automated Electrophoresis System (Bio-Rad, Hercules, CA, USA). RNA-seq libraries were prepared with the NEBNext® Ultra™ II Directional RNA sample preparation kit (New England Biolabs, Inc., Ipswich, MA, USA). Library concentrations were quantified with the Qubit 2.0 Fluorometric Quantitation system (Life Technologies, Carlsbad, CA, USA), and the size distribution was assessed using the Experion Automated Electrophoresis System (Bio-Rad, Hercules, CA, USA). For sequencing, samples were diluted and pooled into NGS libraries in equimolar amounts.

### Next-generation sequencing and raw data acquisition

Expression profiling libraries were sequenced on a HiSeq 3000 instrument (Illumina, San Diego, CA, USA) following a 50-base-pair, single-end recipe. Raw data acquisition (HiSeq Control Software, HCS, HD 3.4.0.38) and base calling (Real-Time Analysis Software, RTA, 2.7.7) was performed on-instrument, while the subsequent raw data processing off the instruments involved two custom programs (https://github.com/epigen/picard/) based on Picard tools (2.19.2) (https://broadinstitute.github.io/picard/). In a first step, base calls were converted into lane-specific, multiplexed, unaligned binary alignment map (BAM) files suitable for long-term archival (IlluminaBasecallsToMultiplexSam, 2.19.2-CeMM). In a second step, archive BAM files were demultiplexed into sample-specific, unaligned BAM files (IlluminaSamDemux, 2.19.2-CeMM).

### Transcriptome analysis

Next-generation sequencing (NGS) reads were mapped to the Genome Reference Consortium GRCm38 assembly via “Spliced Transcripts Alignment to a Reference” (STAR, 2.7.9a) [83] utilising the “basic” Ensembl transcript annotation from version e100 (April 2020) as reference transcriptome. Since the mm10 assembly flavour of the University of California, Santa Cruz (UCSC) Genome Browser was preferred for downstream data processing with Bioconductor packages for entirely technical reasons, Ensembl transcript annotation had to be adjusted to UCSC Genome Browser sequence region names. STAR was run with options recommended by the ENCODE project. NGS read alignments overlapping Ensembl transcript features were counted with the Bioconductor (3.14) GenomicAlignments (1.30.0) package via the summarizeOverlaps function in Union mode, ignoring secondary alignments and alignments not passing vendor quality filtering. Since the NEBNext® Ultra™ II Directional RNA protocol leads to sequencing of the first strand, all alignments needed inverting before strand-specific counting in feature (i.e., gene, transcript, and exon) orientation. Transcript-level counts were aggregated to gene-level counts, and the Bioconductor DESeq2 (1.34.0) [84] package was used to test for differential expression based on a model using the negative binomial distribution.

An initial exploratory analysis included principal component analysis (PCA), multidimensional scaling (MDS), sample distance, and expression heatmap plots, all annotated with variables used in the expression modelling (ggplot2 [85], 3.3.6, and Bioconductor ComplexHeatmap [86], 2.10.0), as well as volcano plots (Bioconductor EnhancedVolcano [87] , 1.12.0). Biologically meaningful results were extracted from the model, and log2-fold values were shrunk with the CRAN ashr [88] (2.2.-54) package, while two-tailed *p*-values obtained from Wald testing were adjusted with the Bioconductor Independent Hypothesis Weighting [89] (IHW, 1.22.0) package.

### Gene set enrichment analysis

GSEA was performed using the hallmark data sets from the Molecular signatures database v7.4 (MSigDB, Broad Institute; http://software.broadinstitute.org/gsea/msigdb) and the GSEA software version 2.4.3 [90, 91]. Additional GSEA was performed for an acinar-specific NF-κB gene set. Genes upregulated with a significant fold change of least 2 from [64] were chosen to generate the reference gene list (Additional file 2: Supplementary Table 1). Immune cell gene sets were derived from [65] (Additional file 2: Supplementary Table 2). Significant enrichments were defined false discovery rate <0.25.

### Putative TBX3-DNA binding analysis

All the analysis was performed using R and Bioconductor (R Core Team, 2022). *Lef1* promoter sequence was retrieved from M. musculus UCSC genome version mm10 known Gene database. The position weight matrix (PWM) was generated using the 8 entries of *Lef1* promoter regions and was subsequently used for the sequence logo. Mouse TBX3 palindromic binding site was obtained from Uniprot (https://www.uniprot.org/uniprotkb/P70324/entry). Sequence logos, relative similarity score, correlation, Euclidean distance, and *p*-value were calculated using Transcription Factors binding sites tools in R.

### Statistical analysis

Statistical analysis was performed with GraphPad Prism 9.3.1 software. Data are expressed as individual data points and mean ± SEM (indicated in figure legends). Significance levels for islet size, insulin+, and glucagon+ cells were calculated with a Mann-Whitney test. Significance was evaluated with a two-way ANOVA and a Sidak post-test for the investigation of number of ADM per field, edema score, infiltration, score, proliferation, and fibrosis in the acute pancreatitis model. Significance levels for apoptosis, *Acta2* gene expression, and immune cell infiltrations at 72h after onset of the pancreatitis were assessed by Mann-Whitney test. Significance levels were defined as the following: *p* < 0.05 = *, *p* < 0.01 = **.

## Supplementary Information

**Additional file 1: Fig. S1.** Expression patterns of specific marker genes for cluster assignment in murine pancreata. **Fig. S2. ***TBX3* is expressed during human pancreatic differentiation and in stellate cells of the adult pancreas. **Fig. S3.** Antibody and co-expression validation of TBX3 and Venus markers and validation of pancreatic recombination. **Fig. S4.** TBX2 expression in *Ptf1a-Cre* and *Nkx3-2-Cre* driven *Tbx3*-KO mice pancreata. **Fig. S5. ***TBX3*-knockdown does not impair the formation of human pancreatic tissue. **Fig. S6. ***Tbx3* depletion does not alter T cell and macrophage infiltration during tissue regeneration after acute pancreatitis.

**Additional file 2: Supplementary Table 1.** Upregulated acinar-specific NF-KB response geneset. **Supplementary Table 2.** Immune cell gene sets. **Supplementary Table 3.** Antibodies for histology.

**Additional file 3.** Includes individual values for quantitative data depicted in Figs 3, 4 and 5.

**Additional file 4.** Includes individual values for quantitative data depicted in Supplementary Figures 2 and 6.

## Data Availability

All data generated or analysed during this study are included in this published article, its supplementary information files, and publicly available repositories. Quantitative data generated in this study are presented in Additional file 3 and Additional file 4. The newly generated raw data from RNA-seq can be accessed from the Gene Expression Omnibus repository via the accession code GSE216889 (https://www.ncbi.nlm.nih.gov/geo/query/acc.cgi?acc=GSE216889). GSE101099, GSE109774, GSE162547, and GSE109774 were accessed for data analysis through the Gene Expression Omnibus. Other data will be made available upon reasonable request.

## Abbreviations

ADM: Acinar-to-ductal metaplasia
BAM: Binary alignment map
DEG: Differentially expressed genes
E: Embryonic day post conception
Epi: Epithelial
FCS: Fetal calf serum
FFPE: Formalin-fixed paraffin-embedded
FPKM: Fragments per kilobase of transcript per million mapped reads
GSEA: Gene set enrichment analysis
HM: Hallmark
iPSC: Induced pluripotent stem cells
MDS: Multidimensional scaling
Mes: Mesenchymal
MPO: Myeloperoxidase
NGS: Next-generation sequencing
P: Postpartum day after birth
PCA: Principal component analysis
PUB: Porcine urinary bladder
PWM: Position weight matrix
shRNA: Small hairpin RNA
STAR: Spliced Transcripts Alignment to a Reference
Tbx3: T-box 3
UCSC: University of California, Santa Cruz
UMAP: Uniform manifold approximation and projection
