# Supplementary material for: TBX3 is dynamically expressed in pancreatic organogenesis and fine-tunes regeneration
Source: BMC Biol. 2023 Mar 20;21:55. doi: 10.1186/s12915-023-01553-x (PMC10029195; doi:10.1186/s12915-023-01553-x)
Supplement: Supplementary file 1 — Additional file 1: Fig. S1. Expression patterns of specific marker genes for cluster assignment in murine pancreata. Fig. S2. TBX3 is expressed during human pancreatic differentiation and in stellate cells of the adult pancreas. Fig. S3. Antibody and co-expression validation of TBX3 and Venus markers and validation of pancreatic recombination. Fig. S4. TBX2 expression in Ptf1a-Cre and Nkx3-2-Cre driven Tbx3-KO mice pancreata. Fig. S5. TBX3-knockdown does not impair the formation of human pancreatic tissue. Fig. S6. Tbx3 depletion does not alter T cell and macrophage infiltration during tissue regeneration after acute pancreatitis. [file 12915_2023_1553_MOESM1_ESM.zip › Additional_File_Figure_Legends_Supplementary_Figures_ESM.docx]

**Supplementary Figure Legends:**

**Supplementary Figure 1. Expression patterns of specific marker genes for cluster assignment in murine pancreata**. **a.** Expression patterns of epithelial (*Epcam*), neural crest (*Tlx2*), endothelial (*Pecam1*), mesenchymal (*Col3a1*) marker genes in embryonic pancreata at E12 (n=2 mice). **b and c.** Expression of acinar (*Cpa1*), ductal (*Sox9*), endocrine (*Chga*), endothelial *(Pecam1*), and mesenchymal (*Col3a1*) marker genes in **b** E14.5(n=3 mice) and **c** E17.5 (n=3 mice) pancreata. **d.** Expression patterns of acinar (*Prss1*), ductal (*Sox9*), beta (*Ins1*), alpha (*Gcg*), delta (*Sst*), endothelial (*Pecam1*), stellate (*Col3a1*) cell markers in adult pancreata. UMAP, uniform manifold approximation and projection. Expression levels depicts log-normalized counts.

**Supplementary Figure 2.** ***TBX3* is expressed during human pancreatic differentiation and in stellate cells of the adult pancreas**. **a.** Schematics of a 13-day human pluripotent stem cell differentiation protocol toward pancreatic progenitor cells. **b.** Bulk RNA-expression analysis of different *T-box* genes in embryonic stem cells, definitive endoderm cells, pancreatic endoderm cells, and pancreatic progenitor cells based on data from (29,30, GSE131817) (n≥5). Values depict column-wise Z-scores with clustering based on the Ward.D2 method. **c.** UMAP cell-cluster representation of a re-analyzed single-cell RNAseq dataset for a pancreatic ductal differentiation from human pluripotent stem cells (GSE162547). The lower plot indicates *TBX3* expression throughout different cell populations. **d.** Bulk RNA-expression analysis of different *TBX3* interaction partner genes in embryonic stem cells, definitive endoderm cells, pancreatic endoderm cells, and pancreatic progenitor cells based on data from (29,30). Values depict row-wise Z-scores with clustering based on the Ward.D2 method. **e.** UMAP plot shows single-cell transcriptomes from GSE84133 in the adult pancreas (n=4 donors). The upper panel indicates cellular identity as confirmed by marker expression (**f**), the lower panel indicates *TBX3* expression in different cell populations. **f.** Expression patterns of acinar (*CPA1*), ductal (*KRT19*), beta (*INS*), alpha (*GCG*), delta (*SST*), PP (*PPY*), endothelial (*PECAM1*), stellate (*COL3A1*) cell markers in adult pancreata from **e**. UMAP, uniform manifold approximation and projection. Expression levels of single-cell RNAseq depicts log-normalized counts.

**Supplementary Figure 3. Antibody and co-expression validation of TBX3 and Venus markers and validation of pancreatic recombination. a.** Schematics of the analyzed *Tbx3^wt/wt^* (E14.5) and *Tbx3^Cre/Cre^* (E13.5) embryos for antibody validation. Representative immunofluorescent stainings of mesenchymal TBX3-expression by TBX3 (green), CDH1 (red), and DNA (DAPI, blue) in the *Tbx3^wt/wt^* (left) and *Tbx3^Cre/Cre^* (right) embryos (n=1 mouse per genotype). **b.** Schematics of the analyzed *Tbx3^+/Ven^* mouse strain. Representative immunofluorescent stainings of TBX3 (left, green) or Venus (anti-GFP, right, green), CDH1 (red), and DNA (DAPI, blue) in seminal vesicles of the *Tbx3^+/Ven^* mouse strain (n=1 mouse per genotype). **c.** IHC-staining of TBX3 in the seminal vesicle (left) and an E18.5 pancreas of the *Tbx3^+/Ven^* mouse strain (n=1 mouse). **d** Representative immunohistochemical staining for tdRFP by an mCherry antibody in one control mouse and one *Ptf1a*-Cre^+/-^ x *LSL-tdRFP^KI/KI^* mouse (n=1 mouse per genotype) (55,56). Specific recombination by tdRFP staining (anti-mCherry) is indicated. Scale bars indicate 50 µm.

**Supplementary Figure 4.** **TBX2 expression in *Ptf1a-Cre* and *Nkx3-2-Cre* driven *Tbx3*-KO mice pancreata.** **a.** Representative IHC staining of TBX2 in n=4 control (littermates of *Tbx3-*KO (epi)) pancreata and n=3 *Ptf1a-Cre* driven *Tbx3-*KO (epi) pancreata. **b.** Representative IHC staining of TBX2 in n=5 control (littermates of *Tbx3-*KO (mes)) pancreata and n=4 *Nkx3-2-Cre* driven *Tbx3-*KO (mes) pancreata. **d** Scale bars indicate 50 µm.

**Supplementary Figure 5. *TBX3*-knockdown does not impair the formation of human pancreatic tissue.** **a.** Schematics representing the experimental setup of a porcine urinary bladder (PUB) organ culture model to investigate iPSC-derived pancreatic progenitors and their potential to form all three lineages (ductal, acinar, endocrine cells) of the pancreas with respect to the doxycycline-inducible expression of an anti-*TBX3* shRNA. **b.** H&E stainings of grafts derived from pancreatic progenitor cells after 2 weeks on the PUB. **c.** Ductal marker (KRT19) expression in iPSC-derived ductal structures on the PUB. **d.** Acinar marker (TRY, trypsin) expression in iPSC-derived acinar cells on the PUB. **e.** Endocrine (beta cell) marker (INS, insulin) expression in iPSC-derived endocrine cells on the PUB. *TBX3*-wildtype (wt), left; *TBX3*-knockdown (KD), right. Scale bars indicate 100 µm. n=2 independent experiments.

**Supplementary Figure 6.** ***Tbx3* depletion does not alter T cell and macrophage infiltration during tissue regeneration after acute pancreatitis.** **a.** Immunohistochemistry staining for CD3+ T cells at 72h after induction of acute pancreatitis. **b.** Quantification of CD3+ T cells per field of view in n=9 control (littermates of *Tbx3-*KO (epi)) pancreata and n=9 *Ptf1a-Cre* driven *Tbx3-*KO (epi) pancreata. **c.** Immunohistochemistry staining for F4/80+ macrophages at 72h after induction of acute pancreatitis. **d.** Quantification of F4/80+ macrophages per field of view in n=8 control (littermates of *Tbx3-*KO (epi)) pancreata and n=9 *Ptf1a-Cre* driven *Tbx3-*KO (epi) pancreata. Graphs display individual data points, mean and SEM. Mann-Whitney-U-test was performed to assess significance levels (not significant). Scale bars represent 100 µm.
