# Supplementary material for: TBX3 is dynamically expressed in pancreatic organogenesis and fine-tunes regeneration
Source: BMC Biol. 2023 Mar 20;21:55. doi: 10.1186/s12915-023-01553-x (PMC10029195; doi:10.1186/s12915-023-01553-x)
Supplement: Supplementary file 2 — Additional file 2: Supplementary Table 1. Upregulated acinar-specific NF-KB response geneset. Supplementary Table 2. Immune cell gene sets. Supplementary Table 3. Antibodies for histology. [file 12915_2023_1553_MOESM2_ESM.docx]

**Supplementary Table 1: Upregulated acinar-specific NF-KB response geneset** (64)

| *CD40* |
| --- |
| *MMP9* |
| *CSF2RB* |
| *CD83* |
| *CCL5* |
| *CSF2* |
| *CSF3* |
| *PTGS2* |
| *CCND1* |
| *SELP* |
| *CDKN1A* |
| *PDGFB* |
| *AKT1* |
| *MAP2K6* |
| *NR4A2* |
| *BCL2L1* |

**Supplementary Table 2: Immune cell gene sets** (65)

| **B_cells** | **T_cells** | **Macrophages** | **Monocytes** | **Neutrophils** | **NK_cells** | **Plasma_cells** |
| --- | --- | --- | --- | --- | --- | --- |
| *AFF3* | *AMICA1* | *ADAMDEC1* | *AGTRAP* | *ACSL1* | *KIR2DL1* | *GUSBP11* |
| *BANK1* | *APBB1IP* | *ADORA3* | *AIF1* | *ALPK1* | *KIR2DL2* | *IGH* |
| *BLK* | *ARHGAP15* | *AOAH* | *C10orf54* | *AQP9* | *KIR2DL3* | *IGHG3* |
| *BTLA* | *ARHGAP25* | *ARRB2* | *CD14* | *BASP1* | *KIR2DL4* | *IGJ* |
| *CCR6* | *ARHGAP9* | *ATP8B4* | *CD300LF* | *BCL6* | *KIR2DL5A* | *IGKC* |
| *CD180* | *BIN2* | *BCL2A1* | *CD33* | *CD97* | *KIR2DS1* | *IGKV1D-13* |
| *CD19* | *BTK* | *C1orf54* | *CD93* | *CEP19* | *KIR2DS2* | *IGLC1* |
| *CD22* | *C1orf162* | *C1QA* | *CTSD* | *CFLAR* | *KIR2DS3* | *IGLJ3* |
| *CD37* | *CCL19* | *C1QB* | *EMILIN2* | *CSF3R* | *KIR2DS5* | *IGLL3P* |
| *CD72* | *CCR7* | *C2* | *FCN1* | *CXCR2* | *KIR3DL1* | *IGLVA* |
| *CD79A* | *CD2* | *C3AR1* | *FES* | *DENND5A* | *KIR3DL2* | *IGLV1-44* |
| *CD79B* | *CD27* | *C5AR1* | *FGR* | *DYSF* | *KIR3DL3* | *MZB1* |
| *CR2* | *CD28* | *CCR1* | *GNS* | *FAM65B* | *KLRC2* | *TNFRSF17* |
| *EBF1* | *CD3D* | *CCRL2* | *GRN* | *FCGR2C* | *KLRC3* | *TXNDC5* |
| *FAM129C* | *CD3E* | *CD163* | *HCK* | *FPR1* | *KLRC4* |  |
| *FCRL1* | *CD3G* | *CD300A* | *HMOX1* | *GLT1D1* | *KLRD1* |  |
| *FCRL2* | *CD48* | *CD4* | *KIAA0930* | *GPR97* | *PRF1* |  |
| *FCRL3* | *CD52* | *CD68* | *LILRA6* | *IFITM2* | *SAMD3* |  |
| *FCRL5* | *CD6* | *CD74* | *LILRB2* | *IL17RA* | *SH2D1B* |  |
| *FCRLA* | *CD8A* | *CD86* | *LILRB3* | *KCNJ2* | *TBX21* |  |
| *HLA-DOB* | *CD96* | *CECR1* | *LRRC25* | *KIAA0247* |  |  |
| *IGHV5-78* | *CORO1A* | *CLEC7A* | *LST1* | *LILRA2* |  |  |
| *KIAA0125* | *CRTAM* | *CMKLR1* | *NFAM1* | *LIMK2* |  |  |
| *LINC00926* | *CXCL9* | *CSF1R* | *NOTCH2* | *LINC01002* |  |  |
| *LOC100507616* | *CXCR6* | *CTSB* | *PILRA* | *MGAM* |  |  |
| *LY9* | *CYTIP* | *CTSS* | *PLXDC2* | *MOB3A* |  |  |
| *MS4A1* | *DOCK10* | *CYBB* | *PRAM1* | *NAMPT* |  |  |
| *P2RX5* | *DOCK2* | *CYTH4* | *PSAP* | *NCF4* |  |  |
| *PAX5* | *DOCK8* | *DPYD* | *PYCARD* | *PADI2* |  |  |
| *PNOC* | *DPEP2* | *EMR2* | *RHOG* | *PHC2* |  |  |
| *POU2F2* | *EVI2A* | *FCER1G* | *SERPINA1* | *PHF21A* |  |  |
| *S1PR4* | *EVI2B* | *FCGR1A* | *SLC7A7* | *PLXNC1* |  |  |
| *SNX22* | *FAM26F* | *FCGR1B* | *TGFBI* | *PREX1* |  |  |
| *STAP1* | *FLI1* | *FCGR2A* | *THEMIS2* | *RALB* |  |  |
| *TCL1A* | *FYB* | *FCGR3B* | *TIMP2* | *RNF149* |  |  |
| *TLR10* | *FYN* | *FPR3* | *TPP1* | *S100A8* |  |  |
| *VPREB3* |  |  |  |  |  |  |

**Supplementary Table 3: Antibodies for histology**

| **Antibody** | **Species** | **Dilution** | **Application** | **Company** | **Catalogue #** | **RRID** |
| --- | --- | --- | --- | --- | --- | --- |
| **Primary antibodies** | | | | | | |
| AMY2A | rabbit | 1:100 | IF-FFPE | Sigma-Aldrich | A8273-1VL | N/A |
| B220 | rat | 1:40 | IHC-FFPE | BD | 550286 | AB_393581 |
| CD3E | rabbit | 1:150 | IHC-FFPE | Invitrogen | MA5-14524 | AB_10982026 |
| CD49f | rat | 1:300 | IF-Cryo | BD | 555734 | AB_2296273 |
| CK-19 (KRT19) | rat | 1:35 | IF-FFPE | DSHB | 2133570 | N/A |
| CK-19 (KRT19) | mouse | 1:100 | IHC-FFPE | DAKO | M0888 | AB_2234418 |
| Collagen IV | rabbit | 1:400 | IF-Cryo | Abcam | ab6586 | AB_305584 |
| E-Cadherin/CDH1 | mouse | 1:1000/1:2000 | IF-FFPE, IF-Cryo | BD | 610181 | AB_397580 |
| F4/80 | rabbit | 1:5000 | IHC-FFPE | Cell Signaling | 70076 | AB_2799771 |
| FOXA2 | goat | 1:1000 | IF-Cryo | Santa Cruz | sc-6554 | N/A |
| GFP | rabbit | 1:500/1:1500 | IF-Cryo, IF-FFPE | Life Technologies | A6455 | AB_221570 |
| GFP | chicken | 1:1000 | IF-Cryo | Aves Labs | GFP-1020 | AB_10000240 |
| Glucagon, GCG | guinea pig | 1:600 | IF-Cryo | Takara | M182 | AB_2619627 |
| Glucagon, GCG | mouse | 1:1000 | IF-FFPE | Sigma-Aldrich | G2654 | AB_259852 |
| Insulin, INS | rabbit | 1:5000 | IF-FFPE | Abcam | ab181547 | AB_2716761 |
| Insulin, INS | guinea pig | 1:400 | IF-Cryo | Bio-Rad | 5330-0104G | AB_1605150 |
| KI-67 | rabbit | 1:100 | IHC-FFPE | Invitrogen | MA5-14520 | AB_10979488 |
| Laminin | rabbit | 1:500 | IF-Cryo | Sigma-Aldrich | L9393 | AB_477163 |
| mCherry | rabbit | 1:500 | IHC-FFPE | Abcam | ab167453 | AB_2571870 |
| MPO | goat | 1:1000 | IHC-FFPE | R&D Systems | 3667 | AB_2250866 |
| N-Cadherin/ CDH2 | mouse | 1:1000 | IF-Cryo | BD | 610920 | AB_2077527 |
| NKX6-1 | goat | 1:300 | IF-Cryo | R&D Systems | AF5857 | AB_1857045 |
| Pancreatic alpha amylase | rabbit | 1:400 | IF-Cryo | Abcam | ab21156 | AB_446061 |
| PDX1 (D59H3) | rabbit | 1:300 | IF-Cryo | Cell Signaling | 5679 | AB_10706174 |
| PECAM-1 | rat | 1:500 | IF-Cryo | BD | 553370 | AB_394816 |
| TBX2 | Rabbit | 1:200 | IHC-FFPE | Bioss/Thermofisher | BS-0507R | AB_10857458 |
| TBX3 | rabbit | 1:250 | IF-FFPE | Abcam | ab99302 | AB_10861059 |
| TRY (trypsin) | mouse | 1:50 | IHC-FFPE | Santa Cruz | sc-137077 | AB_2300318 |
| TurboGFP | rabbit | 1:250 | IF-FFPE | Thermo Fisher Scientific | PA5-22688 | AB_2540616 |
| Vimentin, VIM | rabbit | 1:200 | IF-Cryo | Abcam | ab92547 | AB_10562134 |
|  |  |  |  |  |  |  |
| **Secondary Antibodies** | | | | | | |
| anti-chicken IgY-Cy2 | donkey | 1:800 | IF-Cryo | Dianova/Jackson | 703-225-155 | AB_2340370 |
| anti-goat IgG 488 | donkey | 1:500 | IF-FFPE | Invitrogen | **A-11055** | AB_2534102 |
| anti-goat IgG 555 | donkey | 1:800 | IF-Cryo | Invitrogen | **A-21432** | AB_2535853 |
| anti-goat IgG 568 | donkey | 1:500 | IF-FFPE | Invitrogen | **A-11057** | AB_2534104 |
| anti-guinea pig 649 | donkey | 1:800 | IF-Cryo | Dianova/Jackson | 706-495-148 | N/A |
| anti-guineapig Cy3 | donkey | 1:800 | IF-Cryo | Dianova/Jackson | 706-165-148 | AB_2340460 |
| anti-mouse IgG 488 | donkey | 1:500 | IF-FFPE | Invitrogen | **A-21202** | AB_141607 |
| anti-mouse IgG Cy5 | donkey | 1:800 | IF-Cryo | Dianova/Jackson | 715-175-151 | AB_2619678 |
| anti-rabbit IgG 488 | donkey | 1:500,  1:800 | IF-FFPE, IF-Cryo | Invitrogen | A-21206 | AB_2535792 |
| anti-rabbit IgG 555 | donkey | 1:800 | IF-Cryo | Invitrogen | A31572 | AB_162543 |
| anti-rabbit IgG 568 | donkey | 1:500 | IF-FFPE | Invitrogen | **A10042** | AB_2534017 |
| anti-rabbit IgG 647 | donkey | 1:800 | IF-Cryo | Dianova/Jackson | 711-605-152 | AB_2492288 |
| anti-rabbit IgG 647 | donkey | 1:500 | IF-FFPE | Invitrogen | **A-31573** | AB_2536183 |
| anti-rat IgG 488 | goat | 1:500 | IF-FFPE | Invitrogen | **A-11006** | AB_2534074 |
| anti-rat IgG 647 | donkey | 1:800 | IF-Cryo | Dianova/Jackson | 712-605-150 | AB_2340693 |
| anti-rat IgG 647 | goat | 1:500 | IF-FFPE | Invitrogen | **A48265** | AB_2895299 |
| anti-rat IgG Cy3 | donkey | 1:800 | IF-Cryo | Dianova/Jackson | 712-165-153 | AB_2340667 |
