# Supplementary material for: TBX3 is dynamically expressed in pancreatic organogenesis and fine-tunes regeneration
Source: BMC Biol. 2023 Mar 20;21:55. doi: 10.1186/s12915-023-01553-x (PMC10029195; doi:10.1186/s12915-023-01553-x)
Supplement: Supplementary file 1 — Additional file 1: Fig. S1. Expression patterns of specific marker genes for cluster assignment in murine pancreata. Fig. S2. TBX3 is expressed during human pancreatic differentiation and in stellate cells of the adult pancreas. Fig. S3. Antibody and co-expression validation of TBX3 and Venus markers and validation of pancreatic recombination. Fig. S4. TBX2 expression in Ptf1a-Cre and Nkx3-2-Cre driven Tbx3-KO mice pancreata. Fig. S5. TBX3-knockdown does not impair the formation of human pancreatic tissue. Fig. S6. Tbx3 depletion does not alter T cell and macrophage infiltration during tissue regeneration after acute pancreatitis. [file Additional_File_1_Figure_S1.pdf › Additional_File_1_Figure_S1.pdf]

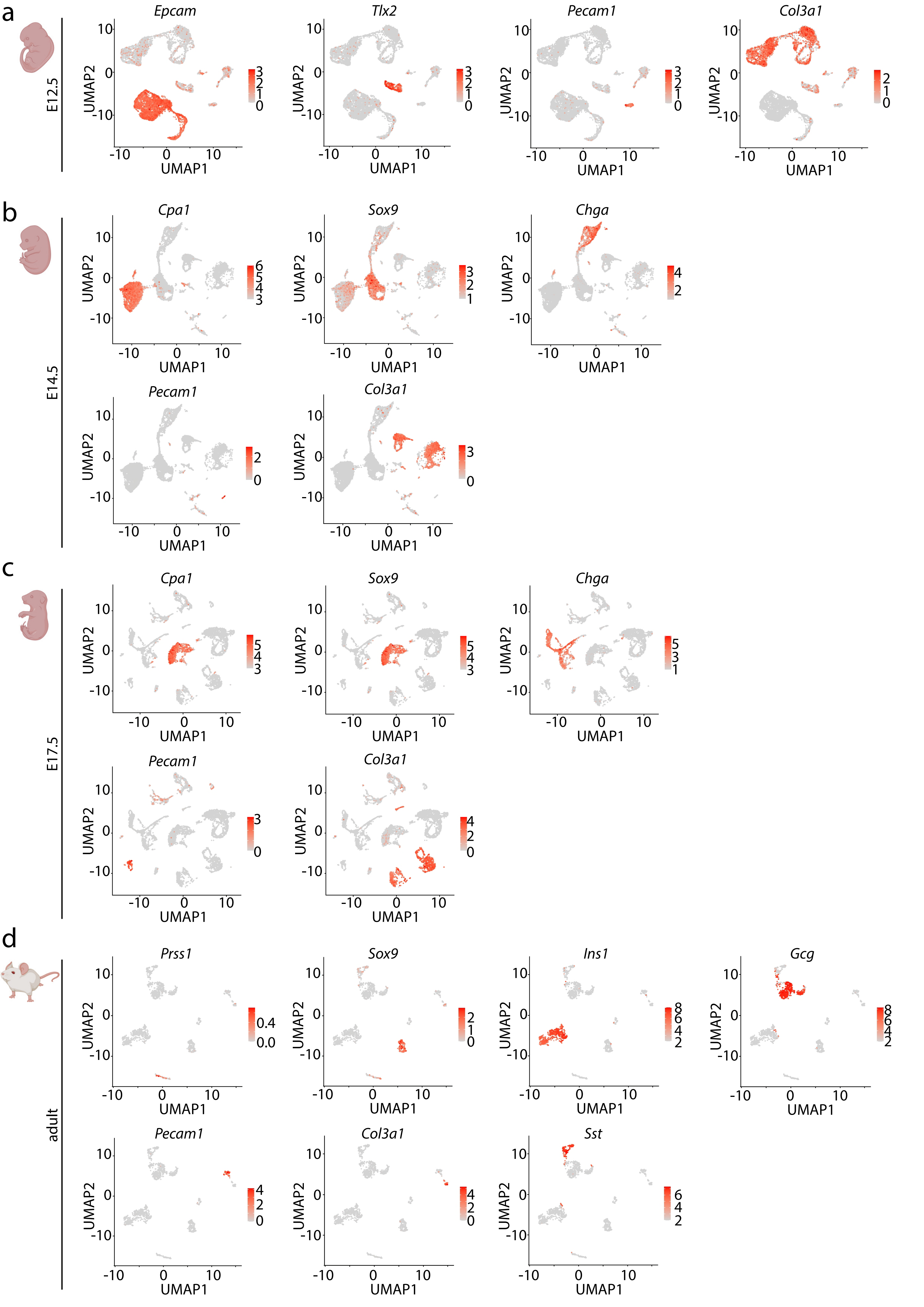

a
E12.5
Epcam
Tlx2
Pecam1
Col3a1
b
E14.5
Cpa1
Sox9
Chga
Pecam1
Col3a1
c
E17.5
Cpa1
Sox9
Chga
Pecam1
Col3a1
d
adult
Prss1
Sox9
Ins1
Gcg
Pecam1
Col3a1
Sst
UMAP1
UMAP2
